# Web-Based Immersive Patient Simulator as a Curricular Tool for Objective Structured Clinical Examination Preparation in Surgery: Development and Evaluation

**DOI:** 10.2196/10693

**Published:** 2018-07-04

**Authors:** Seung-Hun Chon, Sabrina Hilgers, Ferdinand Timmermann, Thomas Dratsch, Patrick Sven Plum, Felix Berlth, Rabi Datta, Hakan Alakus, Hans Anton Schlößer, Christoph Schramm, Daniel Pinto dos Santos, Christiane Bruns, Robert Kleinert

**Affiliations:** ^1^ Department of General, Visceral and Cancer Surgery University Hospital of Cologne Cologne Germany; ^2^ Faculty of Medicine University of Cologne Cologne Germany; ^3^ Department of Gastroenterology and Hepatology University Hospital Cologne Cologne Germany; ^4^ Department of Radiology University Hospital of Cologne Cologne Germany

**Keywords:** immersive patient simulator, simulator-based curriculum, Objective Structured Clinical Examination, artificial learning interface

## Abstract

**Background:**

Objective Structured Clinical Examination is a standard method of testing declarative and process knowledge in clinical core competencies. It is desirable that students undergo Objective Structured Clinical Examination training before participating in the exam. However, establishing Objective Structured Clinical Examination training is resource intensive and therefore there is often limited practice time. Web-based immersive patient simulators such as ALICE (Artificial Learning Interface of Clinical Education) can possibly fill this gap as they allow for the training of complex medical procedures at the user’s individual pace and with an adaptable number of repetitions at home. ALICE has previously been shown to positively influence knowledge gain and motivation.

**Objective:**

Therefore, the aim of this study was to develop a Web-based curriculum that teaches declarative and process knowledge and prepares students for a real Objective Structured Clinical Examination station. Furthermore, we wanted to test the influence of ALICE on knowledge gain and student motivation.

**Methods:**

A specific curriculum was developed in order to implement the relevant medical content of 2 surgical Objective Structured Clinical Examination stations into the ALICE simulator framework. A total of 160 medical students were included in the study, where 100 students had access to ALICE and their performance was compared to 60 students in a control group. The simulator performance was validated on different levels and students’ knowledge gain and motivation were tested at different points during the study.

**Results:**

The curriculum was developed according to the Kern cycle. Four virtual clinical cases were implemented with different teaching methods (structured feedback, keynote speech, group discussion, and debriefing by a real instructor) in order to consolidate declarative and process knowledge. Working with ALICE had significant impact on declarative knowledge gain and Objective Structured Clinical Examination performance. Simulator validation was positive for face, content, construct, and predictive validity. Students showed high levels of motivation and enjoyed working with ALICE.

**Conclusions:**

ALICE offers Web-based training for Objective Structured Clinical Examination preparation and can be used as a selective didactic intervention as it has positive effect on knowledge gain and student motivation.

## Introduction

OSCE (Objective Structured Clinical Examination) is a well-established method used in clinical education. OSCE aims to simulate clinical scenarios to test different core competencies such as physical examination, communication, clinical reasoning, medical interventions, and knowledge of medical procedures [1]. In each OSCE station, students are given an assignment that tests at least one part of a predefined core competency. Performance is evaluated in an individual assessment by an experienced clinical physician.

Effective preparation for an OSCE requires learning and repetition of both declarative (“what to do”) and process (“how to do it”) knowledge. Less complex medical procedures such as resuscitation are usually trained on mannequin simulators [2]. More complex procedures such as clinical decision-making and workflows in diagnosis and therapy require small group training on standardized patients in a simulated OSCE. However, simulation of a complex OSCE situation with tutors, doctors, and standardized patients is resource intensive. Hence, OSCE simulation of complex procedures to prepare for the exam is often not part of the curriculum or occurs only with limited practice time. Since the aim of OSCE is to prepare students for future clinical work, it is desirable that they are given the opportunity to train for the OSCE without time pressure and limits to the number of repetitions before they enter the exam.

Immersive patient simulators (IPS) can potentially fill this gap. IPS are Web-based software programs which allow repetitive training of medical procedures in a virtual environment [3]. With IPS, students can practice complex medical procedures at their own individual pace and with a suitable number of repetitions, even on their computers at home. In a recent study, we developed a proprietary IPS, ALICE (“Artificial Learning Interface of Clinical Education”), and proved it had a positive impact on clinical decision-making and student motivation [4].

In this study, we wanted to develop a simulator-based curriculum that imparts both declarative and process knowledge to prepare students for a real OSCE station. In the next step, we aim to implement this curriculum and the necessary features into ALICE and validate the new ALICE version. Finally, in order to test the hypothesis that ALICE is a useful tool for effective OSCE preparation, we wanted to measure students’ knowledge gain when working with ALICE and test the acceptance of this educational tool.

## Methods

### Adaption of Medical Content for ALICE

For this experiment, we chose a classic surgical OSCE station from trauma surgery. It presents patients with a trauma diagnosis (fracture of distal radius) in different clinical cases.

The underlying disease was added to the ALICE simulator framework as a blueprint. The creation of medical content and programming of additional ALICE features were completed according to the steps of Kern's curriculum planning cycle [5].

**Figure 1:**
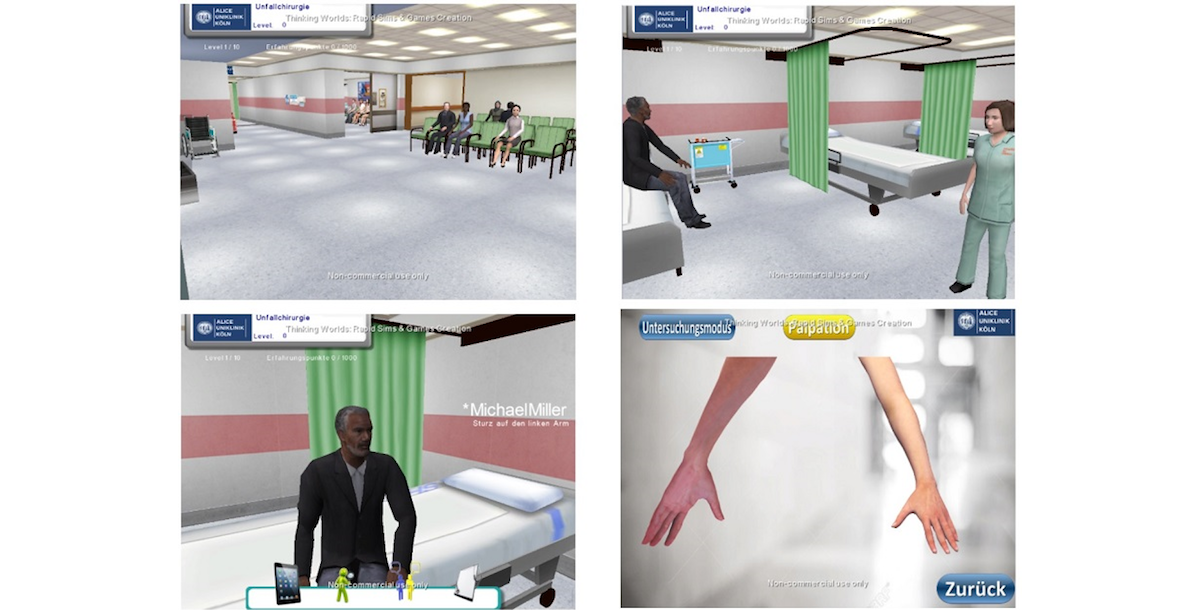
Impressions of ALICE (Artificial Learning Interface of Clinical Education) where students navigate freely and treat virtual patients.

The design and technical realization of ALICE have previously been described by our group [6]. In short, ALICE is a Web-based immersive patient simulator which allows students to navigate through a virtual “game like” environment from a first-person perspective (Figure 1).

ALICE simulates a treatment room with a simulated patient. The user can interact with the virtual patient and additional non-player characters such as nurses and other doctors. The simulator starts with a short introduction teaching the user basic simulator controls and usage of the simulator. The user is able to freely interact with the environment and treat the virtual patient. The user is free to choose between all available tests and there is no restriction on specific or medically indicated tests. When the student chooses an examination not medically indicated, the test shows a normal finding. The simulation ends when the student chooses a diagnosis and initiates the necessary treatment. ALICE stores the students’ behavior at the server level, logging students’ decisions.

### Group Distribution and Study Design

**Figure 2:**
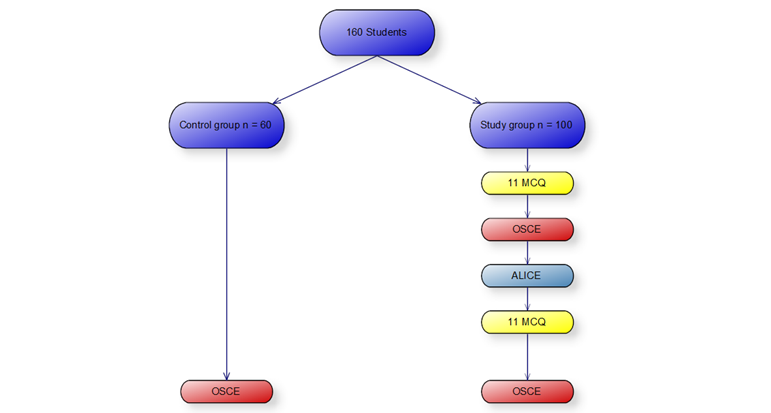
Group distribution of participants; 100 students used ALICE and were compared to a control group with 60 students. MCQ: multiple choice question; OSCE: Objective Structured Clinical Examination.

Participants in the study were 160 medical students. Participation was on a voluntary basis without any financial compensation. The study group was comprised of 100 students and the remaining 60 students were allocated to the control group. The study was approved by the Educational Committee of the Medical Faculty at the University of Cologne. The Institutional Review Board was informed and there were no objections. The impact of ALICE on OSCE performance was tested using a trauma case examined by 100 students. In the first stage of the test, students answered 11 multiple-choice questions (MCQs). They then participated in a real OSCE. The correct results of the MCQs and OSCE were not revealed to the students at this point. In the next stage, the students worked with the simulator (ALICE), after which they repeated the MCQ questionnaire and the OSCE with the same clinical scenario as before (Figure 2). The correct results were then revealed and discussed in a peer-to-peer debriefing and with an experienced doctor.

The control group consisted out of 60 students who prepared for the OSCE without ALICE. All students passed the study completely without any drop outs.

### Validation of ALICE

Declarative knowledge was tested by comparison of students’ pre- and postsimulator performance on the MCQs. Process knowledge was tested by comparison of their pre- and postsimulator OSCE performance.

Process knowledge was measured on two different levels: (1) comparison of treatment of virtual patient 1 versus virtual patient 4 in the study group and (2) comparison of pre- and postsimulator OSCE performance. The following parameters were defined: correct diagnosis, correct therapy, and correct workflow in anamnesis and diagnostics. These workflows were designed as a blueprint reflecting the “optimal” workflow suggested by 2 independent senior surgeons.

Validation of ALICE’s new features and curricular content was tested on different levels according to the “Consensus guidelines for validation of virtual reality surgical simulators” (Table 1) [7].

Face validity was determined by descriptive analysis. Students were asked to judge the degree of resemblance between ALICE and the real OSCE situation. Content validity was defined as the degree to which the system covers the subject matter of the real activity. It was examined by comparison of ALICE performance in the fourth case and with OSCE performance of the control group. Impact of previous knowledge as a degree of discrimination between the different experience levels was tested using a subgroup comparison between students up to third study year and students in fourth year or higher. Predictive validity as sign of impact on future performance was tested by comparison between the OSCE and control group.

**Table 1:** Processes used to test validity of ALICE.

Quality and validity		Method	
**Knowledge gain**			
	Declarative knowledge		Comparison: pre- and post-MCQs^a^
	Procedural knowledge		Comparison: pre- and post-OSCE^b^
**ALICE^c^ validity**			
	Face validity		Similarity study and real activity
	Content validity		Comparison: ALICE - OSCE performance
	Construct validity		Comparison: 3^rd^ year and 4^th^ year students
	Predictive validity		Comparison: OSCE study and control group

^a^MCQ: multiple-choice question.

^b^OSCE: Objective Structured Clinical Examination.

^c^ALICE: Artificial Learning Interface of Clinical Education.

Student acceptance and their opinion about the effectiveness, applicability, and impact on motivation were determined by means of a questionnaire using a (forced choice) 6-point Likert scale (1=extremely satisfied, 2=very satisfied, 3=somewhat satisfied, 4=somewhat dissatisfied, 5=very dissatisfied, 6=extremely dissatisfied).

Data were analyzed with the SPSS Statistics Version 25 (IBM Corp, Armonk, NY, USA) for Windows (Microsoft Corp, Redmond, WA) and Microsoft Excel Version 2013 for Windows (Microsoft Corp, Redmond, WA). Group comparisons were conducted using *t* tests and *P* ⩽.05 was considered statistically significant.

### Group Distribution

The participants in the study were 160 medical students (31 males, 129 females; mean age 27 (SD 2.5) years, age range 21-34 years) who participated in the study on a voluntary basis with no financial compensation. All students were in the clinical phase of their studies (up to third study year n=57; fourth study year and above n=103) and had passed the first medical exam after 2 years of study. All students were recruited at the University Hospital of Cologne. Students worked on all 4 clinical cases. ALICE was accessible one day before the OSCE.

## Results

### Adaption of Medical Content for ALICE

ALICE’s content was extended by adding diagnostic procedures for trauma patients. Four clinical cases were presented in a firm sequence according to the curricular demands of the specific curricular needs (Table 2). All students worked on all four cases. The framework of medical content was based on the national learning objectives catalogue.

The learning goals were defined and are summarized in Textbox 1.

**Table 2:** Clinical cases added to ALICE for this study.

Patient number	Trauma	
	Diagnosis	Therapy
1	Distal radius fracture (Colles)	Conservative
2	Distal radius fracture (Smith)	Reposition and surgery
3	Distal radius fracture (Colles)	Reposition and surgery
4	Distal radius fracture (Colles)	Conservative

**Learning goals for Objective Structured Clinical Examination (OSCE) intervention.**

After working with ALICE, students should:

- know the underlying basics in anatomy, physiology, pathophysiology and surgery
- assign specific symptoms to corresponding diseases
- identify the symptoms of virtual patients that are specific to a given disease
- weigh pathological findings and bring them into the clinical context
- demonstrate correct workflow in diagnostic and therapeutic decisions (clinical reasoning)
- make the correct diagnosis and choose the correct therapy

### Educational Goals

The simulation itself covers different *teaching methods*. ALICE starts with a short animation which covers simulator usage. The user is asked to explore the surroundings and familiarize themselves with their avatar in the virtual world. After treatment of the first virtual patient, the user is given *structured feedback* and a *keynote speech* from the virtual instructor. These 2 methods allow teaching of both declarative and process knowledge. After finishing the cases, students first have a *group discussion* with other students after which they are finally *debriefed* by a real instructor on the educational level of a medical doctor. These two methods consolidate declarative and process knowledge.

Cognitive Engagement was designed according to the ICAP framework (Interactive, Constructive, Active, and Passive) [8]. Working with the simulator puts students in an active learner role and finding the right diagnostic and therapeutic workflow is constructive. Virtual instructors accompanied the students during their cases in the sense of a virtual cognitive apprenticeship. Instructors reduced their support step-by-step according to the number of correct clinical decisions and correct diagnoses made by the students.

### Evaluation

#### Declarative Knowledge

**Figure 3:**
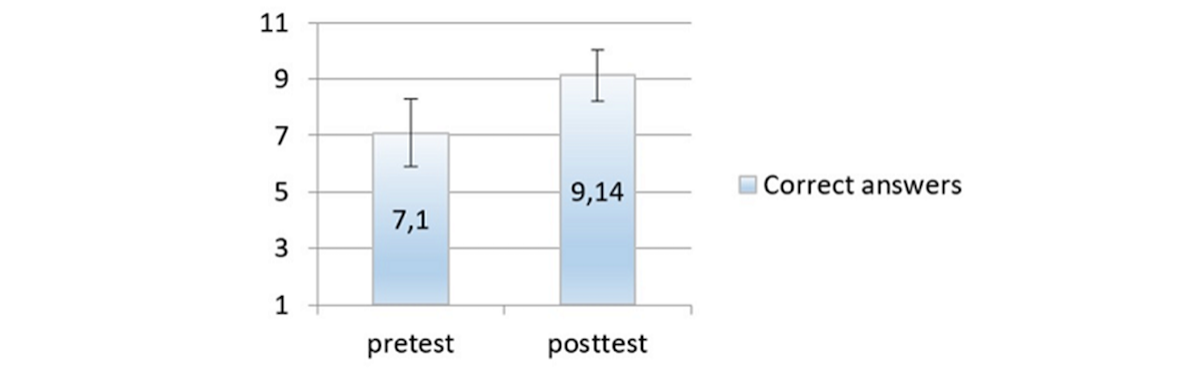
Increase in multiple-choice question (MCQ) scores showing working with ALICE had a positive impact on declarative knowledge.

The influence of simulator use on declarative knowledge was measured by comparing the students’ results in the pre- and postsimulator questionnaire for the 100 students in the study group. The number of correct answers increased significantly between the pre- and postsimulator questionnaires. The mean scores for the pre- and postsimulator questionnaires were 7.1 (SD 1.1) and 9.14 (SD 0.8; *P*=.009) respectively (Figure 3). Therefore, working with ALICE had a positive impact on declarative knowledge.

#### Process Knowledge

**Figure 4:**
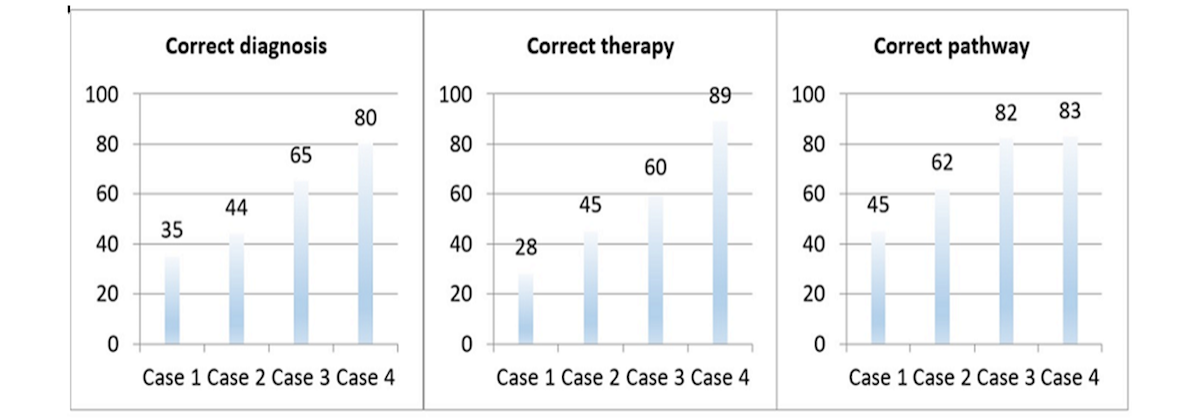
Working with ALICE had a positive impact on students’ process knowledge (Y axis shows number of students).

Students significantly increased their performance from case 1 to case 4 on all 3 indicators of process knowledge, namely correct diagnosis, treatment, and diagnostic pathway (*P=*.002). Therefore, working with ALICE had a positive impact process knowledge (Figure 4).

**Figure 5:**
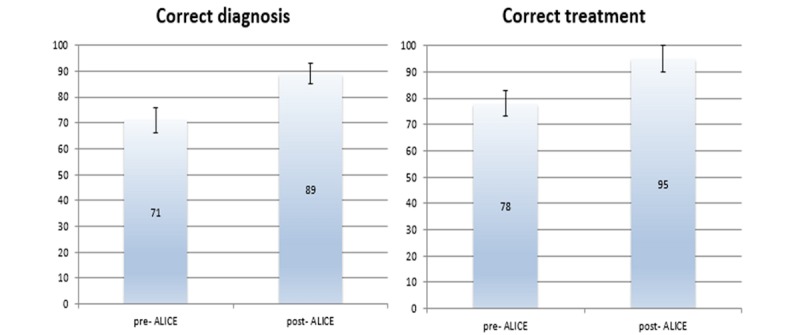
Working with ALICE led to a significant increase in process knowledge (Y axis shows number of students).

The effect of ALICE on OSCE performance was tested by comparing performance on the trauma OSCE before and after working with ALICE (Figure 5). Again, working with ALICE led to a significant increase in process knowledge (*P*=.004).

#### Face Validity

**Figure 6:**
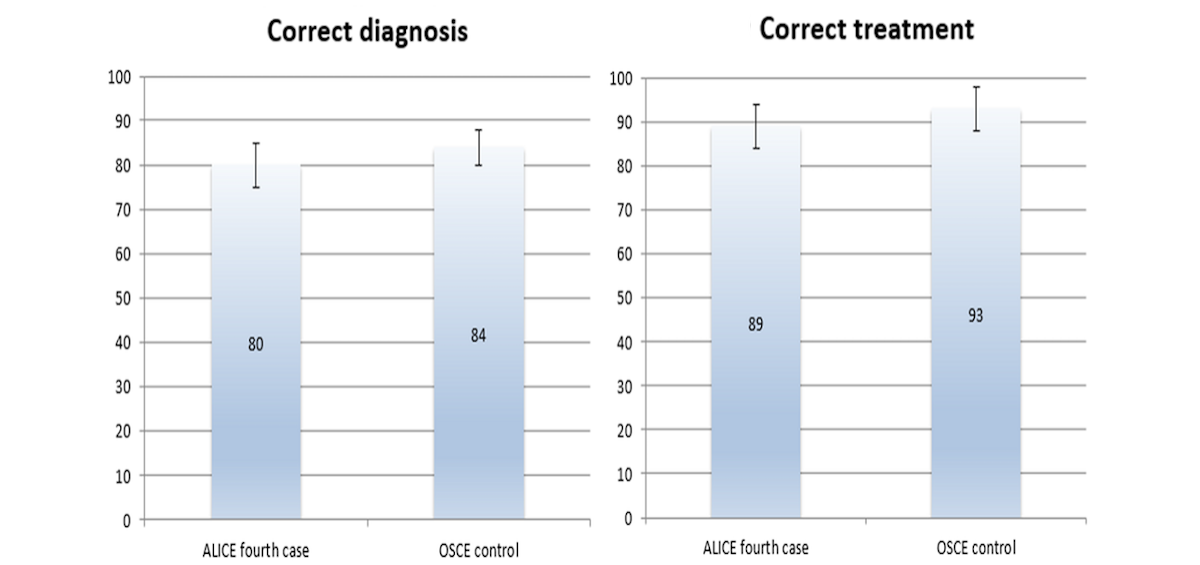
Comparison of ALICE performance in the fourth case and Objective Structured Clinical Examination (OSCE) performance of the control group (Y axis shows number of students).

In a descriptive questionnaire, students judged the degree of resemblance between simulated OSCE in ALICE and the real OSCE situation. As shown in Figure 6, most of the students supported the hypothesis that ALICE can prepare for OSCE as it represents a virtual OSCE situation.

#### Content Validity

Comparison of ALICE performance in the fourth case and OSCE performance of the control group revealed, that there was no difference (*P*=.45) between these groups, proving that there is positive content validity (Figure 6).

#### Construct Validity

Students in their eighth semester and above performed significantly better in finding the correct diagnosis (mean correct diagnosis 94 [SD 6] vs 78 [SD 4]; *P*=.04) and correct treatment (mean correct treatment 98 [SD 7] vs 84 [SD 6]; *P*=.03) in the post-ALICE OSCE compared to the younger students. Therefore, ALICE also had positive construct validity.

#### Predictive Validity

Comparison of post-OSCE performance in the ALICE group and OSCE performance in the control group revealed no statistical difference between the two groups. Therefore, ALICE has positive predictive validity as it is comparable to conventional OSCE preparation (mean correct diagnosis 89 [SD 5] vs 84 [SD 11]; *P*=.42 and mean correct treatment 95 [SD 7] vs 93 [SD 8]; *P*=.56).

**Figure 7:**
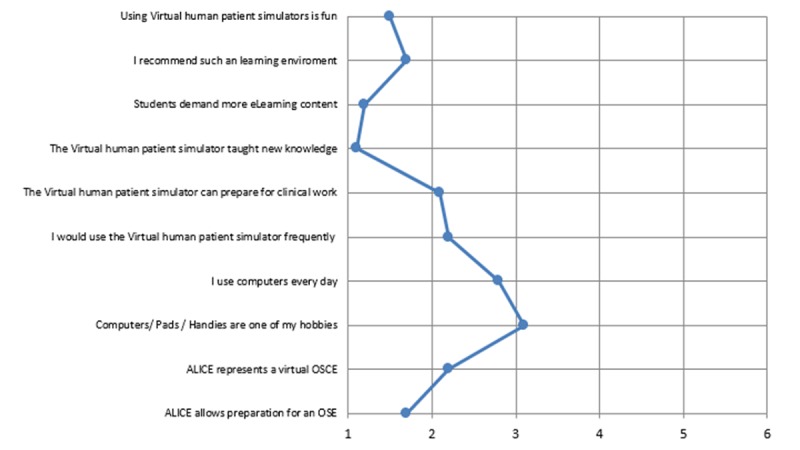
Likert scale (1=extremely satisfied, 2=very satisfied, 3=somewhat satisfied, 4=somewhat dissatisfied, 5=very dissatisfied, 6=extremely dissatisfied) revealed that students felt that ALICE represented a virtual Objective Structured Clinical Examination (OSCE) situation and can help them prepare for an OSCE.

The questionnaire data also revealed a high level of student motivation when working with ALICE. Students enjoyed using ALICE and recommended working with such a simulator to prepare for an OSCE. They felt they learned new topics and demanded more interactive content in their curriculum. The majority of the students would use such a simulator frequently and believe that IPS can help prepare them for future work. The overall impression of the simulator was positive. Interestingly, students showed a normal relationship with technology as the proportion of casual users to power users (students that have computers as a hobby and use them every day) was normal (Figure 7).

## Discussion

### Principal Findings

The current study reveals that ALICE is a suitable tool for OSCE preparation. It showed that ALICE has validity (content, construct, and predictive) and leads to a positive impact on knowledge gain. It is inevitable that each modification of such a simulator requires internal validity testing as major changes may affect the effect of such a curricular intervention.

ALICE was designed as a low-cost 3D immersive framework which makes it possible to add different clinical cases in a Web-based scenario with learning that is not time or location dependent. This enables learning at the individual’s own pace and with the number of repetitions appropriate for them. Differences in knowledge levels can be evened out and future performance can potentially be raised. However, IPS can only be used as a support for a real OSCE training in terms of a blended learning concept since the degree of reality created by these simulators nowadays is not yet of suitable quality.

Moreover, IPS are limited to simulating clinical decision-making based on defined guidelines or clinical blueprints. In this current feasibility study, we used a comparatively simple OSCE station, namely interpretation of X-rays and a limited number of possible clinical decisions enables a steep learning curve [9]. More complex scenarios, however, are technically possible [10] but their impact on OSCE performance has not yet been proven for these modules. Furthermore, these educational tools are often used as an additional tool for preparation. A study that compares students who used ALICE exclusively for preparation of a complex case and students who prepared with conventional learning methods is desirable and part of future studies. However, the current study reflects reality, as most learners do not rely on only one knowledge source when preparing for an examination.

ALICE shows a positive impact on learners’ motivation and both their declarative and procedural knowledge. However, these findings may be influenced by the fact that participation in this study was on a voluntary basis which is known to possibly bias the result as motivated students more often agree to participation than less motivated students [11]. It is important to note that learners have different learning styles and thus not all learners are equally responsive to this educational tool [12]. Hence, the results cannot be generalized for all students.

Although ALICE stored user data, there are little information about the time spent on the simulator and the number of repetitions. ALICE logged the decisions and corresponding time points, however there are no information about students’ behavior like breaks, restarts, or distractions. There was no “full logging” of students’ behavior such as “time logged on,” IP address etc as these program algorithms would require routines similar to spyware, and such implementation was out of the question. Therefore, the influence of number of repetitions and time spent on ALICE cannot be answered in this study.

Embedding a simulator such as ALICE in an educational environment requires thorough curricular planning and subsequent implementation of the curriculum into the simulator. This initial investment pays off once the simulator can be used as an alternative to established teaching methods with high running costs. The current student generation puts high requirements on the quality and motivation of their education [13]. Motivation is known to have a strong effect on knowledge gain [14]. ALICE supports motivation by offering exploration of an immersive world with freedom of choices and treatment of the user’s own virtual patients [15]. These factors promote experience of autonomy and competency which can directly affect intrinsic motivation [16]. Extrinsic motivation was enhanced by applying features that are commonly used in video games such as reward systems (badges, points, rank), continuous feedback, and leader boards for students [17]. The use of IPS for psychomotor or communicative learning goals is already described in literature and its effect on knowledge gain in these settings is still not proven. Adding these competences to the ALICE framework is part of future studies.

ALICE was designed to cover all qualities of the ICAP framework: Interactive, Constructive, Active and Passive. Hence ALICE promotes not only knowledge gain but also influences students’ intrinsic motivation [18]. However, ALICE is most effective when used in a blended learning context as the group discussion is highly interactive and this is thought to have a positive effect on learners’ performance [19].

### Conclusion

ALICE is a valuable tool for teaching declarative and process knowledge in a Web-based setting. It shows positive impact on knowledge gain and student motivation and therefore enriches the toolbox of didactic methods for OSCE preparation.

## Appendix

Multimedia Appendix 1 ALICE - Artificial Learning Interface for Clinical Education.

## Abbreviations

ALICE: Artificial Learning Interface of Clinical Education
ICAP: interactive, constructive, active, passive
IPS: immersive patient simulators
MCQ: multiple choice question
OSCE: Objective Structured Clinical Examination
